# Editorial: Emerging talents in molecular innate immunity: 2023

**DOI:** 10.3389/fimmu.2024.1364552

**Published:** 2024-01-12

**Authors:** Francesca Granucci, Uday Kishore

**Affiliations:** ^1^ Department of Biotechnology and Biosciences, University of Milano-Bicocca, Milan, Italy; ^2^ Department of Veterinary Medicine (CAVM), United Arab Emirates University, Al Ain, United Arab Emirates

**Keywords:** innate immunity, young researchers’ contributions, arthropod immunity, signalling, vaccine

In the dynamic and ever-evolving field of Molecular Innate Immunity, there lies a reservoir of untapped potential and emerging talent. The “*Emerging Talents in Molecular Innate Immunity: 2023*” Research Topic represents the innovative work of young scientists who are shaping the future of immunological research. Many young scientists in the realm of Molecular Innate Immunity are engaged in groundbreaking research, pushing the boundaries of our understanding of the immune system. Their work, inherently crucial, often remains cloistered within academic circles, unseen by a broader audience. Recognizing this gap, this initiative from the Frontiers in Immunology seeks to bridge the divide, providing these bright minds a platform to share their findings with the wider scientific community and beyond.

One of the formidable challenges faced by young researchers is the daunting prospect of peer review- a critical yet intimidating step in the journey of scientific publication. Frontiers journals redefine this process as a collaborative endeavor. Their interactive peer-review system is meticulously designed to offer not just evaluation but mentorship, guiding young researchers through constructive feedback, and supportive engagement.

This Research Topic serves a dual purpose. It is both a showcase of innovative research and a launchpad for identifying and fostering emerging leaders in the field. By highlighting these young talents, this Research Topic not only celebrates their current achievements but also invites the community to engage with and follow their burgeoning careers. We invite readers to explore this Research Topic and the fresh perspectives and novel insights offered by the next generation of immunologists.

This Research Topic is composed of 5 articles including both reviews and original research articles.

Perveen et al. explored the innate immune responses of hematophagous arthropods such as mosquitoes and ticks to various pathogens, including viruses. This review article focuses on the insect immune system, particularly haemocytes and their various functions including phagocytosis and encapsulation. Despite their complex immune responses, these vectors often transmit viruses to human and animal hosts. The study underlines the importance of understanding virus-vector-host dynamics and advocates for a multidisciplinary approach to develop new strategies against vector-borne viral infections.

Bonam et al. present a retrospective study to examine the immunostimulatory mechanism of LiteVax™ Adjuvant (LVA), an oil-in-water emulsion vaccine adjuvant. LVA, containing CMS (Maltose 4’-monosulphate 1,2,3,6,2’,3’,6’-heptadecanoic acid ester) and other components, showed efficacy in animal models; however, its exact mechanism remained unclear. The research focused on dendritic cells (DC) that are crucial in adaptive immune response; it revealed that CMS within LVA enhances DC activation markers, cytokine production, and CD4^+^ T cell response, unlike the control. This suggests a unique role of CMS in LVA as an immunostimulatory agent, with LVA serving as a delivery system.

In their original work, Gerogianni et al. focus on Iron oxide nanoparticles (IONPs), commonly used in medicine, which can inadvertently activate the body’s immune response, leading to adverse effects. In this work, the authors show that in blood, IONPs initiate inflammation and activate the complement system, significantly increasing cytokine levels and causing thromboinflammation. Therefore, use of complement inhibitors holds promise in mitigating these unintended immune reactions, thereby enhancing the safety and effectiveness of IONPs in their therapeutic and diagnostic applications.

Another original work was reported by Brescia et al. The work focuses on Th17^+^ cells that are crucial in coordinating innate and adaptive immune responses. Serum and glucocorticoid regulated kinase 1 (SGK1) plays a vital role in controlling Th17^+^ cell development via IL-23R, influencing Forkhead box protein O1 (FOXO1) cellular location through phosphorylation. This study indicates that RAN-binding protein 1 (RANBP1) is a key player in this process, affecting SGK1 functions and Th17^+^ cell maturation, specifically by modulating FOXO1 nuclear transport, which is crucial for the Th17^+^ immune function.

Finally, Zhang et al. focused on the role of cGAMP synthase (cGAS)-stimulator of interferon genes (STING) pathway in pulmonary fibrosis. Pulmonary fibrosis, a deadly lung disease marked by an excessive extracellular matrix production and fibroblast activation, currently lacks a cure. Available FDA-approved drugs only slow down its progression. Recent research emphasizes the significant role of the cGAS-STING pathway in the pathogenesis, involving DNA detection and immune response activation. This review article explores the cGAS-STING pathway’s role in pulmonary fibrosis and the therapeutic potential of targeting this pathway, including using cGAS and STING inhibitors.

In conclusion, the “*Emerging Talents in Molecular Innate Immunity: 2023*” Research Topic shows the remarkable contributions of young scientists to the field of immunology. From exploring the immune responses in arthrpods to the immunostimulatory mechanisms of vaccine adjuvants, in addition to the impact of nanoparticles on the immune system and the critical role of proteins in immune cell development, each article offers valuable insights. Moreover, the investigation into the cGAS-STING pathway’s influence on pulmonary fibrosis underscores the need for novel therapeutic approaches. This Research Topic not only highlights the current achievements of these emerging scientists but also sets the stage for their future activities, which will undoubtedly continue to shape and advance the immunological research.

## Author contributions

FG: Writing – original draft. UK: Writing – review & editing.

